# The complete chloroplast genome sequence of *Phedimus Kamtschaticus* (Crassulaceae) in Korea

**DOI:** 10.1080/23802359.2018.1437819

**Published:** 2018-02-12

**Authors:** Hee-Seung Seo, Seung-Chul Kim

**Affiliations:** Department of Biological Sciences, Sungkyunkwan University, Suwon, Korea

**Keywords:** Chloroplast genome, *Phedimus kamtschaticus*, Crassulaceae, Korea

## Abstract

The complete chloroplast genome sequence of *Phedimus kamtschaticus*, which commonly occurs in northeastern Asia was determined. The genome size was 151,652 bp, composed of one pair of inverted repeats (IRs) of 25,977 bp, which were separated by one large single-copy (LSC; 83,010 bp) and one small single-copy (SSC; 16,688 bp) region. The chloroplast genome contained 132 genes, including 88 protein-coding genes, 36 tRNA genes, and 8 rRNA genes. The overall GC content was 37.8%. Phylogenetic analysis of the complete chloroplast genome suggested that *P. kamtschaticus* was most closely related to Ulleung Island insular endemic *P. takesimensis*.

The family Crassulaceae is a morphologically diverse and taxonomically complex angiosperm family, comprising 35 genera and approximately 1500 species (Berger 1930). While the Crassulaceae are considered a natural group, infrafamilial classification has been extremely difficult due to homoplasious morphological features (van Ham 1995; Soltis et al. 2000). The genus *Phedimus* (ca. 20 species in Asia and Europe) represents one lineage of nonmonophyletic subfamily Sedoideae. Traditionally, species of *Phedimus* have been treated as members of *Sedum*, but recent phylogenetic study strongly supported the monophyly of *Phedimus* and its segregation from *Sedum* (Mayuzumi and Ohba 2004). Of ca. 20 species of *Phedimus*, we selected *P. kamtschaticus*, which occurs commonly in the northeastern Asia, and sequenced its complete chloroplast genome because it is considered to be a continental progenitor of Ulleungdo and Dokdo Island endemic *P. takesimensis* in Korea.

We collected fresh leaves of *P. kamtschaticus* from Mt. Hambaek, Korea (GPS coordinates 37°09′16.6″N 128°54′53.4″E; altitude 1334 m above the sea level) and a voucher specimen (SKK014113) was deposited at the herbarium of Sungkyunkwan University (SKK). Total DNA was iso lated using the DNeasy Plant Mini Kit (Qiagen, Carlsbad, CA), following the instructions of the manufacturer. Sequencing was done using the Illumina Miseq (Illumina Inc., San Diego, CA) platform and assembled by SPAdes 3.6.1 (Bankevich et al. 2012) and CLC Genomics Workbench v.5.5.1 (CLC Bio, Aarhus, Denmark). The chloroplast genome of *P. kamtschaticus* was annotated using the Dual Organellar GenoMe Annotator (DOGMA) tool (Wyman et al. 2004) and CpGAVAS (Liu et al. 2012) with plastid/bacterial genetic code. The tRNAs were confirmed using tRNAscan-SE with default settings (Schattner et al. 2005). Gene annotation was done using BLAST X, Geneious v.8.1.6. (Biomatters Ltd., Auckland, New Zealand), and then manually corrected for intron/exon boundaries. The complete chloroplast genome of *P. kamtschaticus* was aligned with 11 representative species of Saxifragales using MAFFT v.7 (Katoh and Standley 2013). Maximum likelihood (ML) analysis was conducted based on 13 complete chloroplast genomes (1 outgroup and 12 ingroup) using IQ-TREE v.1.4.2 (Nguyen et al. 2015) with 1000 bootstrap (BS) replications.

Here, we reported the complete chloroplast genome sequence of *P. kamtschaticus* (MG680403), which has a total length of 151,652 bp with 37.8% GC content, and contained two inverted repeat regions (IRa and IRb) of 25,977 bp, separating a large single-copy (LSC) region of 83,010 bp and a small single-copy (SSC) region of 16,688 bp. The chloroplast genome contained 132 genes, including 88 protein-coding genes, 36 tRNA genes, and 8 rRNA genes. The structure, gene content and order, and GC content of *P. kamtschaticus* were similar to those of *P. takesimensis* chloroplast genome.

The phylogenetic relationship of *P. kamtschaticus* was determined based on 11 other representative chloroplast genomes in Saxifragales. The phylogenetic analysis showed that the family Crassulaceae, including four species of *Phedimus* and *Sedum*, was monophyletic (100% BS) (Figure 1). *P. kamtschaticus* was most closely related to Ulleung Island endemic *P. takesimensis* in Korea, establishing a continental progenitor and insular derivative relationship.

**Figure 1.**
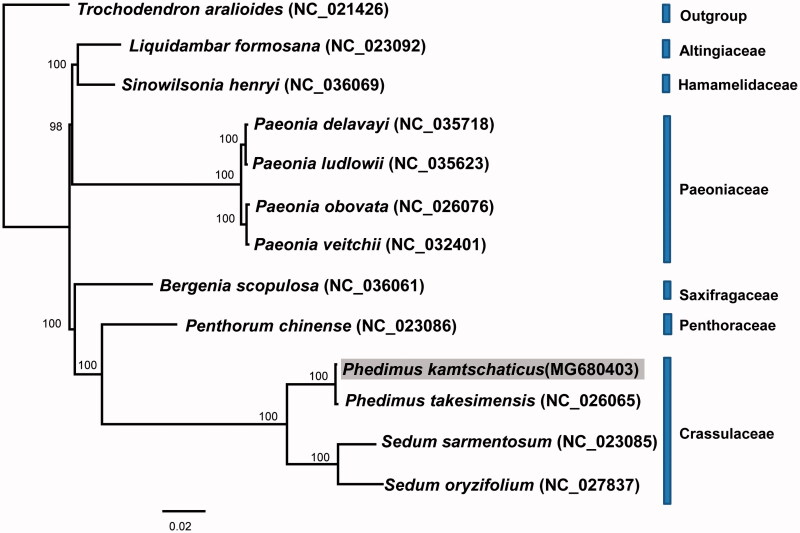
Maximum likelihood tree based on the complete chloroplast genome from 11 representative species of Saxifragales. *Trochodendron aralioides* (Trochodendraceae) was used as an outgroup and the bootstrap support values >50% are shown in the branches.

## References

[CIT0001] Bankevich A, Nurk S, Antipov D, Gurevich AA, Dvorkin M, Kulikov AS, Lesin VM, Nikolenko SI, Pham S, Prjibelski AD, et al 2012 SPAdes: a new genome assembly algorithm and its applications to single-cell sequencing. J Comput Biol. 19:455–477. 22506599 10.1089/cmb.2012.0021 PMC3342519

[CIT0002] Berger A. 1930 Crassulaceae In: Engler A, Prantl K, editors. Die Natürlichen Pflanzenfamilien, Vol. 2, 18a. Leipzigp: W. Engelmann; p. 352–483.

[CIT0003] Katoh K, Standley DM. 2013 MAFFT multiple sequence alignment software version 7: improvements in performance and usability. Mol Biol Evol. 30:772–780. 23329690 10.1093/molbev/mst010 PMC3603318

[CIT0004] Liu C, Shi L, Zhu Y, Chen H, Zhang J, Lin X, Guan X. 2012 CpGAVAS, an integrated web server for the annotation, visualization, analysis, and GenBank submission of completely sequenced chloroplast genome sequences. BMC Genomics. 13:715. 23256920 10.1186/1471-2164-13-715 PMC3543216

[CIT0005] Mayuzumi S, Ohba H. 2004 The phylogenetic position of eastern Asian Sedoideae (Crassulaceae) inferred from chloroplast and nuclear DNA sequences. Syst Bot. 29:587–598.

[CIT0006] Nguyen L-T, Schmidt HA, von Haeseler A, Minh BQ. 2015 IQ-TREE: a fast and effective stochastic algorithm for estimating maximum-likelihood phylogenies. Mol Biol Evol. 32:268–274. 25371430 10.1093/molbev/msu300 PMC4271533

[CIT0007] Schattner P, Brooks AN, Lowe TM. 2005 The tRNAscan-SE, snoscan and snoGPS web servers for the detection of tRNAs and snoRNAs. Nucleic Acids Res. 33:W686–W689. 15980563 10.1093/nar/gki366 PMC1160127

[CIT0008] Soltis DE, Soltis PS, Chase MW, Mort ME, Albach DC, Zanis M, Savolainen V, Hahn WH, Hoot SB, Fay MF, et al 2000 Angiosperm phylogeny inferred from 18S rDNA, *rbcL*, and *atpB* sequences. Bot J Linn Soc. 133:381–461.

[CIT0009] van Ham RCHJ. 1995 Phylogenetic relationships in the Crassulaceae inferred from chloroplast DNA variation In: Hart H’t, Eggli U, editors. Evolution and systematics of the Crassulaceae. Leiden: Blackhuys Publishers; p. 16–29.

[CIT0010] Wyman SK, Jansen RK, Boore JL. 2004 Automatic annotation of organellar genomes with DOGMA. Bioinformatics. 20:3252–3255. 15180927 10.1093/bioinformatics/bth352

